# Phantoms of the forest: legacy risk effects of a regionally extinct large carnivore

**DOI:** 10.1002/ece3.1866

**Published:** 2016-01-15

**Authors:** Ellinor Sahlén, Sonja Noell, Christopher S. DePerno, Jonas Kindberg, Göran Spong, Joris P.G.M. Cromsigt

**Affiliations:** ^1^ Department of Wildlife, Fish, and Environmental StudiesSwedish University of Agricultural SciencesSkogsmarksgrändSE‐901 83UmeåSweden; ^2^ Fisheries, Wildlife and Conservation Biology ProgramDepartment of Forestry and Environmental ResourcesNorth Carolina State University110 Brooks AveRaleighNC27607; ^3^ Department of Integrative Biology and Biodiversity ResearchInstitute for Wildlife Biology and Game ManagementUniversity of Natural Resources and Life SciencesViennaAustria; ^4^ Department of ZoologyCentre for African Conservation EcologyNelson Mandela Metropolitan UniversityPO Box 77000Port Elizabeth6031South Africa

**Keywords:** Anthropogenic change, antipredator response, brown bear, landscape of fear, predator‐prey interactions, prey naivety, ungulates

## Abstract

The increased abundance of large carnivores in Europe is a conservation success, but the impact on the behavior and population dynamics of prey species is generally unknown. In Europe, the recolonization of large carnivores often occurs in areas where humans have greatly modified the landscape through forestry or agriculture. Currently, we poorly understand the effects of recolonizing large carnivores on extant prey species in anthropogenic landscapes. Here, we investigated if ungulate prey species showed innate responses to the scent of a regionally exterminated but native large carnivore, and whether the responses were affected by human‐induced habitat openness. We experimentally introduced brown bear *Ursus arctos* scent to artificial feeding sites and used camera traps to document the responses of three sympatric ungulate species. In addition to controls without scent, reindeer scent *Rangifer tarandus* was used as a noncarnivore, novel control scent. Fallow deer *Dama dama* strongly avoided areas with bear scent. In the presence of bear scent, all ungulate species generally used open sites more than closed sites, whereas the opposite was observed at sites with reindeer scent or without scent. The opening of forest habitat by human practices, such as forestry and agriculture, creates a larger gradient in habitat openness than available in relatively unaffected closed forest systems, which may create opportunities for prey to alter their habitat selection and reduce predation risk in human‐modified systems that do not exist in more natural forest systems. Increased knowledge about antipredator responses in areas subjected to anthropogenic change is important because these responses may affect prey population dynamics, lower trophic levels, and attitudes toward large carnivores. These aspects may be of particular relevance in the light of the increasing wildlife populations across much of Europe.

## Introduction

Although large carnivores are threatened on most continents, these species, along with other large mammals, are currently undergoing a revival in Europe (Enserink and Vogel 2006; Kindberg et al. 2011). In this process, large carnivores are recolonizing landscapes where they have been absent for centuries and that have been heavily modified by humans (Chapron et al. 2014). Research on predator**–**prey interactions has been initiated in North American (Berger 1978; Altendorf et al. 2001; Creel et al. 2005; Beschta and Ripple 2008; Halofsky and Ripple 2008; Lashley et al. 2014) and African ecosystems (Underwood 1982; Valeix et al. 2009; Thaker et al. 2011); however, information about the consequences of carnivores on the behavior and space use of their ungulate prey in heavily human‐modified European landscapes is lacking. Here, we recognize two aspects that we consider particularly relevant; prey naivety toward recolonizing carnivores, and human alterations to the perceived “landscape of fear” (Laundré et al. 2001).

To avoid predation, prey may adjust behavior, morphology, or physiology (Lima 1998; Kunkel and Pletscher 2000; Relyea 2001; Jayakody et al. 2008; Abate et al. 2010; Hossie et al. 2010). Such responses may carry indirect costs by reducing long‐term survival, growth, and reproduction (Boonstra et al. 1998; Laundré et al. 2001; Creel et al. 2007) with population effects potentially exceeding those of direct predation (Creel and Christianson 2008). It has been suggested that prey species may lose their antipredator behavior over time if predators disappear from the system (Sih et al. 2010). Therefore, the losses of carnivores in Europe during the last centuries may have resulted in naive prey that fails to properly respond to predation risk (Berger et al. 2001; Sand et al. 2006). If antipredator behaviors are lost, prey may be more susceptible to predation if predators return (Berger et al. 2001), which may affect interspecific interactions and population dynamics, if only for a transient period. The alternative hypothesis is that prey maintain the ability to recognize their extinct predators for a long time (Li et al. 2011; Chamaillé‐Jammes et al. 2014), perhaps due to an innate fear of predators (Ferrero et al. 2011).

In addition to the possibility of prey naivety, anthropogenic landscape alterations may strongly influence prey responses to predation risk. Landscape features mediate risk (Poysa 1994; Kunkel and Pletscher 2000; Jayakody et al. 2008) and prey may perceive an altered “landscape of fear” (Laundré et al. 2001). In the presence of predators, the predation risk perceived by prey varies depending upon features such as terrain, barriers, and habitat types (Laundré et al. 2001). Habitat openness plays a particularly important role in mediating predator**–**prey interactions, by affecting vigilance levels (Jayakody et al. 2008) and prey distribution (Valeix et al. 2009; Laundré et al. 2010). For example, sites with less horizontal cover have been shown to be perceived as risky areas by elk *Cervus canadensis* and moose *Alces alces* in terms of wolf predation (Kunkel and Pletscher 2000; Creel et al. 2005). However, other studies have failed to detect an effect of habitat openness on antipredator response in closed forest systems with little human impact (Kuijper et al. 2014). Instead they concluded that escape impediments increased perceived predation risk (Kuijper et al. 2013). Anthropogenic landscape change in Europe is extensive, and forestry or agricultural practices have increased the variation of habitat openness in forested landscapes through the construction of forest clear‐cuts and agricultural fields (Estreguil et al. 2013). Such alterations may increase opportunities for ungulates to use the variation in habitat openness to reduce predation risk. Currently, information on antipredator responses and risk effects in human‐modified European landscapes is largely lacking (but see Lone et al. 2014).

Our objective was to explore the antipredator behavior of prey species in a diverse community of European ungulates and assess how habitat openness altered the nature of their responses. To test this, we experimentally introduced the scent of brown bear (a historically native but now locally extinct predator), and a novel nonpredator scent, in a highly human‐modified landscape in southeastern Sweden and evaluated the responses of five sympatric ungulate species.

## Material and Methods

### Study area

**Figure 1:**
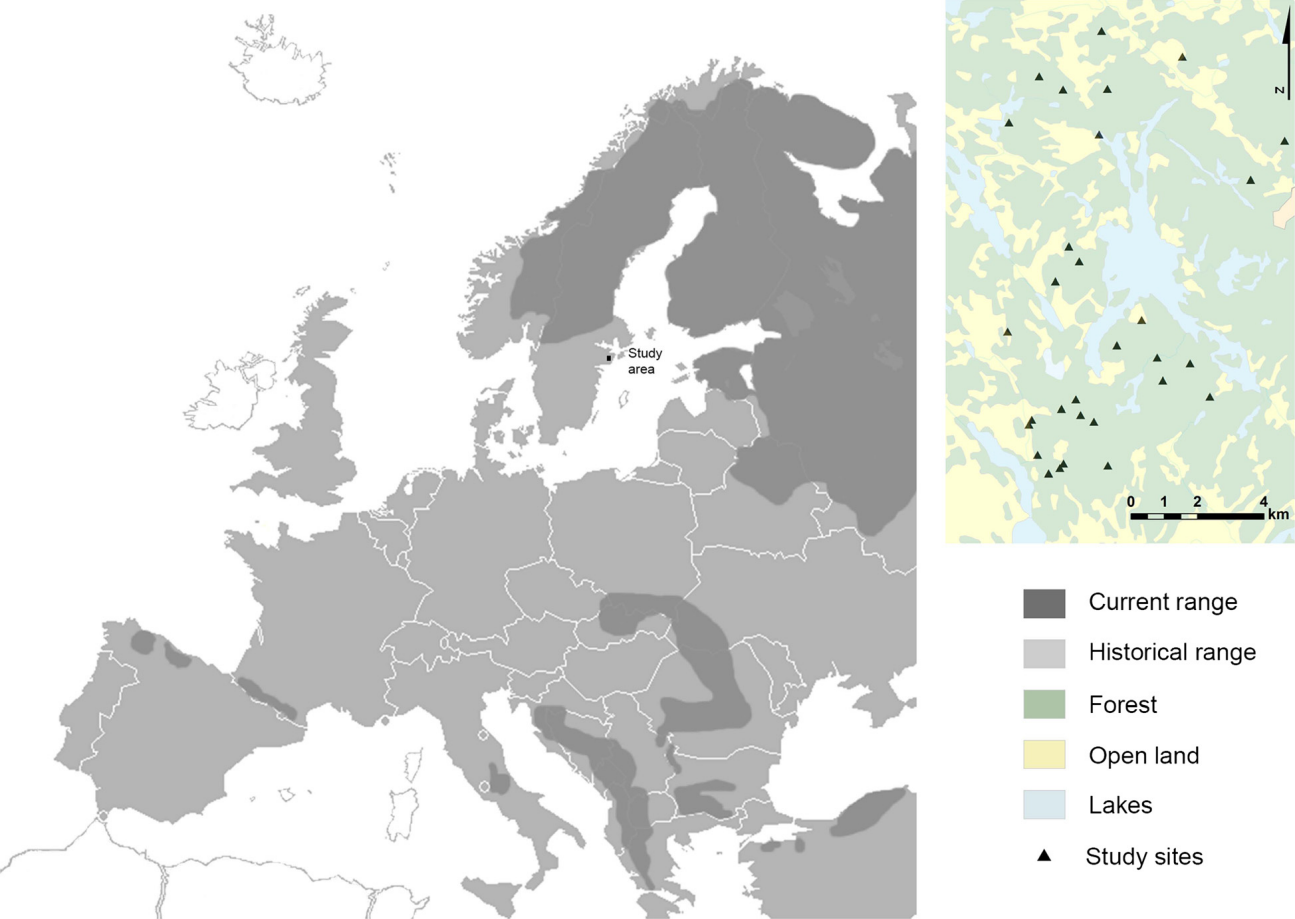
The current and historical distribution of the brown bear in Europe, and the location of the study area (black dot) in southeastern Sweden, in March and April 2013. In Scandinavia, the brown bear is currently expanding from its core areas, where remnant populations subsisted after the end of a long extermination campaign (in early 1900s). The inset map shows the study sites (black pyramids) across the landscape extensively modified by humans (forest converted to open agricultural land).

Because of the absence of large carnivores and high diversity of sympatric ungulate species we conducted our study in Södermanland County in southeastern Sweden (Fig. 1). The landscape is forest dominated but highly fragmented with agricultural lands and clearcuts, which form a heterogeneous patch work of closed forest and open land (see inset in Fig. 1). The forests are mainly composed of boreal coniferous production stands of Scots pine *Pinus sylvestris* and Norway spruce *Picea abies*; however, numerous broad‐leaved tree species occur throughout the study area, including birch *Betula* spp., alder *Alnus* spp., oak *Quercus robur*, rowan *Sorbus aucuparia*), and aspen *Populus tremula*. In Sweden, populations of brown bear and wolf *Canis lupus* are recovering and recolonizing their historic ranges. Both species were eradicated from the study area over 170 years ago (Statistics Sweden 1984; Swenson et al. 1995) and have not returned. The outer range of the nearest established brown bear population was 100–150 km away from the study area and the nearest wolf pack >50 km (SEPA 2015). Bear sightings in the vicinity of the study area are rare (>10 years ago). The occurrence of wolves wandering through the study area is likely slightly higher. Most ungulates in the study area are unlikely to have ever encountered a bear or a wolf. The sympatric ungulate species in the study area include roe deer *Capreolus capreolus*, red deer *Cervus elaphus*, fallow deer *Dama dama*, moose *A. lces alces*, and wild boar *Sus scrofa*. All species are native to Sweden, except fallow deer, which was introduced in Sweden in the 1570s as a game species from its original Holocene distribution in the Mediterranean and Persia (Dolman and Waber 2008). Relative abundance estimates indicate that fallow deer constitute 61% of the ungulate community, wild boar 23%, roe deer 8%, red deer 5%, and moose 3% (Öster Malma 2013). Red fox *Vulpes vulpes* and lynx *Lynx lynx* occur in the area (although the latter is rare). Both species can predate on ungulates, especially fawns. Predator scent as such is thus not novel to the ungulates in the area.

We conducted the study during spring 2013 (March 6–April 19). The coldest temperatures occurred in March (min: −18.4°C, max: 8.4°C) and became successively warmer at the onset of spring during April (min: −9.4°C, max: 14.1°C). However, temperatures remained relatively low throughout most of our study period, resulting in a packed, icy snow crust covering the ground. For more than 5 years, extensive supplemental feeding of ungulates have occurred throughout the study area during winter and early spring, with ungulates readily using these sites and accustomed to human disturbance and scent. Landowners distributed silage to feeding sites in the area throughout the course of our study period (due to the persistently cold weather).

### Study design

We used camera traps to document ungulate visitation to 30 artificial feeding sites in forested habitats. The same type of feed (wheat silage) was used at all sites, and feed piles (bales) were distributed by local landowners across an area of roughly 25 m^2^ at each site. All feeding sites had access roads that were used by humans in the area as thoroughfare, for recreation, and distribution of feed. The study was conducted outside of hunting season, and feeding sites were never close to a settlement (>1 km away). The influence of direct human activity on our results would have been minimal. Each site was exposed to three weekly scent treatments conducted in a block design including brown bear scent, reindeer scent, and controls. We used the reindeer scent treatment as a noncarnivore novel scent; reindeer are confined to northern Sweden and do not occur in the study area (>250 km away). Hence, each site hosted all three treatments during three consecutive weeks (1 week per treatment). We chose this design so that we could easily correct for site effects. Logistics did not allow us to monitor all 30 sites at the same time. Therefore, we conducted the experiment in two rounds; 15 sites were monitored for 3 weeks during March 6–March 27 and 15 sites for 3 weeks during March 28–April 19. During each week, equal amount of sites were exposed to the three different treatments: five exposed to brown bear scent, five to reindeer scent, and five without scent. Additionally, we arranged the treatments so the nearest sites would have different treatments during the same week.

Apfelbach et al. (2005) suggested that fur has longer lasting effects on prey behavior than feces or urine. Brown bears deposit scent from sebaceous and apocrine glands by rubbing trees, which is used for chemical communications with conspecifics (Clapham et al. 2013), and may function as scent cues for prey species. We mimicked scent‐marking by brown bear by attaching pieces of bear pelt to trees. We used pieces of fresh pelts from wild brown bears (provided by the National Veterinary Institute, Sweden) and reindeer (provided by an anonymous Sami reindeer herder) to introduce carnivore and noncarnivore novel scents into our study area. The pelts were cut into pieces (15 × 15 cm) and nailed to small 15 × 15 cm removable wooden plates, with a 10 × 15 cm “roof” that reduced the effects of snow, rain, and ice. To retain their scent, the pieces of pelt were kept in a freezer (−20°C) until used in the field. Also, low temperatures counteracted decay of pelt pieces. The scent structures were attached at breast height to tree trunks using metal wire. Two scent structures (5–7 m apart) were used at each site, one on each side of a camera and distributed feed (silage), to increase the scent and decrease the effect of wind direction at the sites. Wooden structures of the exact same design but without pieces of fur were attached the same way at sites during control weeks. To maintain similar conditions each week, we exchanged all pelt pieces with fresh pieces for each treatment week, and scent structures were not mixed between treatments. Due to the cold temperatures during our experiment, pelt pieces did not rot. At each site, a remote camera (Scoutguard, model SG560C; HCO Outdoor Products, Norcross, CA) was mounted, directly facing the feed at a distance of 5–10 m. Cameras recorded a 30‐sec video on detection (maximum detection range was 22 m) followed by a 2‐min time lapse during which the camera could not be triggered. The time lapse setting decreased the chance of recording the same individual multiple times, and saved camera battery life. The camera was always positioned so that direct sunlight into the camera lens was avoided and feed centered in the pictures.

Feeding sites had similar forested habitat types (coniferous dominated) but varied in degree of habitat openness. Variation was mainly created by anthropogenic opening of the forests, such as creation of agricultural fields and clear‐cutting practices. Therefore, some feeding sites were surrounded by closed forest, while others were closer to fields or other forest openings (see inset map in Fig. 1). To determine the effect of habitat openness on ungulate visitation, we measured sighting distance at each site using a red and white colored plank (180 cm high, 10 cm wide), which was placed at the feeding structure during measurements (see Ordiz et al. 2009). We measured the distance for the device to be completely hidden as we walked away from it in all four cardinal directions. The average of the four distances (i.e., sighting distance) was used in analyses (DePerno et al. 2003; Ordiz et al. 2009).

### Statistical analyses

We used generalized linear mixed effect models in R (R Core Team 2012) using the MASS package (Ripley et al. 2015) with the quasipoisson family to model use of feeding sites in the study area. Moose and red deer numbers were omitted from all statistical modeling because of few records (*N* < 40). In addition to scent treatment, sighting distance was used as a covariate to investigate the effects of habitat openness at feeding sites and its interaction with the scent treatment. Due to the quasi‐likelihood estimation, we were not able to use likelihood ratio tests for variables used in models. We assessed significant differences between scent treatments using multiple comparison tests (Tukey's) in the multicomp package (Hothorn et al. 2008). We used the number of visits (i.e., the number of times the camera was triggered) as response variables in separate models for each ungulate species.

We modeled the number of visits on a weekly scale, because each site had a constant value of sighting distance (and each treatment lasted for 1 week). The number of visits at feeding sites was summed for each treatment week for each site. All models (three in total) included site and order of treatments as random effects to account for differences in variation among sites and to account for possible effects of treatment order by site.

## Results

The total number of visits to sites varied among species but reflected the relative species abundances in the area: moose and red deer were the least frequent species, roe deer intermediate, and fallow deer and wild boar the most frequent species. All ungulates, except moose (only 14 visits), had more visits to control sites without scent than to brown bear and reindeer scent treatments (Table 1).

**Table 1:** Number of visits (i.e., number of recorded videos) to artificial feeding sites for five sympatric ungulate species in southeastern Sweden, March and April 2013. The number of visits for each species and treatment level represents the number of videos summed over 30 sites and 1 week of sampling per site (i.e., a total of 210 camera trapping days for each species and treatment level combination)

Species	Scent treatment	Visits
Fallow deer	Brown bear	324
	No scent (control)	462
	Reindeer (control)	355
Moose	Brown bear	6
	No scent (control)	1
	Reindeer (control)	7
Red deer	Brown bear	12
	No scent (control)	37
	Reindeer (control)	6
Roe deer	Brown bear	120
	No scent (control)	196
	Reindeer (control)	97
Wild boar	Brown bear	377
	No scent (control)	393
	Reindeer (control)	343

**Figure 2:**
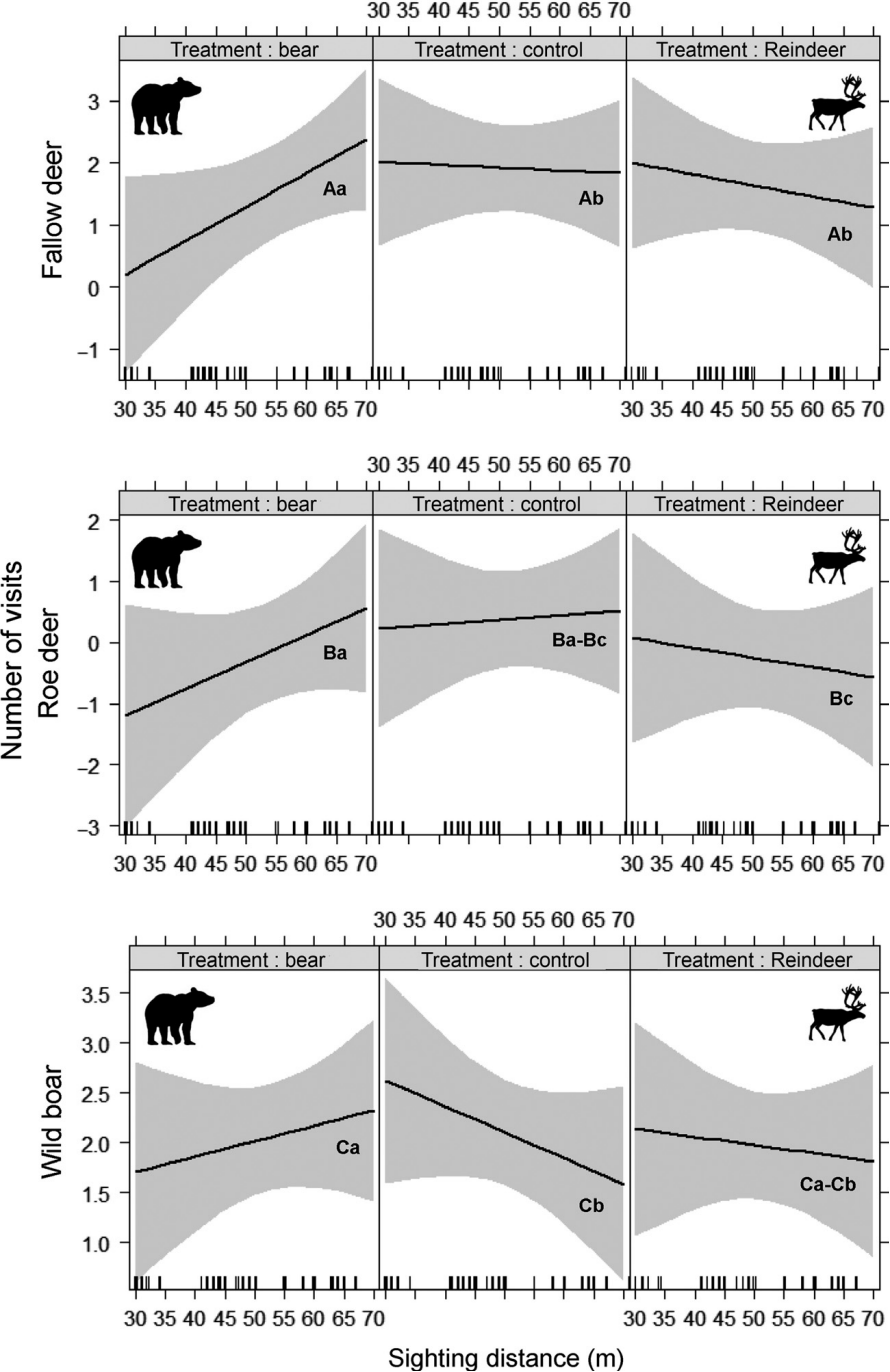
Model estimates of the number of weekly visits and sighting distance (habitat openness) for the three different treatments; brown bear scent (left), no scent (middle), and reindeer scent (right), for fallow deer, roe deer, and wild boar, in southeastern Sweden, March and April 2013. The grey zones represent confidence intervals and the letter coding below the slopes show significance between slopes (e.g., Ba is significantly different to Bc but not to another Ba or Ba‐Bc).

All species were affected by bear and reindeer scents to varying extents, either through lower number of visits or by altering use in response to habitat openness. Fallow deer strongly avoided bear scent compared to other scent treatments (Table 2). In addition, fallow deer showed a positive relationship between the number of visits and sighting distance at sites with bear scent, compared to control and reindeer scent sites where the relationship was negative (Fig. 2). Roe deer did not show clear responses to the introduced scents (Table 2); however, roe deer used sites with bear scent more if these sites were in more open areas (Fig. 2). Wild boar used sites exposed to bear scent less than sites without scent (Table 2) with a positive relationship between the number of visits and sighting distance at sites with bear scent, compared to control sites where the relationship was the opposite (Fig. 2).

**Table 2:** Tukey's multiple comparisons of model estimates for the frequency of ungulate visits to feeding sites with three different scent treatments, in southeastern Sweden, March and April 2013

Ungulate species	Treatment comparison	Estimate	SE	*z*‐value	*P*‐value
Fallow deer	Control – Bear	3.601	1.194	3.015	0.007^a^
	Reindeer – Bear	3.988	1.281	3.113	0.005^a^
	Reindeer – Control	0.387	1.038	0.373	0.926
Roe deer	Control – Bear	2.542	1.352	1.881	0.143
	Reindeer – Bear	3.074	1.527	2.013	0.108
	Reindeer – Control	0.532	1.283	0.415	0.909
Wild boar	Control – Bear	2.155	0.911	2.365	0.047^a^
	Reindeer – Bear	1.136	0.953	1.192	0.458
	Reindeer – Control	−1.019	0.872	−1.169	0.471

a Significant pair‐wise comparison with P < 0.05.

## Discussion

We determined that ungulates avoided predator scent and that perceived risk was mediated by habitat openness. Predator scent avoidance was particularly clear for fallow deer, both in terms of numbers of visits to sites and altered use of habitat openness. At a sighting distance of approximately 60 m the visitation in areas with predator scent approached that of control areas without scent. Our results indicate the human‐created variation in habitat openness in forest landscapes creates opportunities for prey species to change their habitat selection to mediate predation risk. Studies in Białowieża Primeval Forest (BPF), Poland, similar to the one we present here, did not detect an interaction between habitat openness and predator scent for red deer (Kuijper et al. 2014) and roe deer (Wikenros et al. 2015). However, the sighting distance in BPF ranged from 5 to 20 m (compared to 30–70 m in our study), which may be too narrow for ungulates to adjust their habitat selection to predation risk (Kuijper et al. 2013). Hence, instead of ungulates reducing their visitation rate to sites in dense habitats (as we showed in our human‐modified system), ungulates in BPF increased their vigilance and reduced visitation duration to plots with carnivore scent. Whether ungulates select open or closed habitats in the presence of predators may depend on the hunting strategy of the predator (Thaker et al. 2011). Ambush predators are more likely to kill in denser habitat types (Lone et al. 2014), which may push prey into open habitat, whereas the risk of cursorial predators is higher in open areas, resulting in ungulates selecting denser cover (Creel et al. 2005). Brown bears are more likely to occupy relatively dense or rugged habitat (Martin et al. 2010; Ordiz et al. 2011) and may hunt in an ambush‐predatory manner (Garneau et al. 2007), which may be the reason why ungulates in our study reduced their use of sites with dense vegetation when bear scent was present. In addition to the aspect of how far ungulates can see at sites, scent cues may be stronger in denser sites due to less wind, which may intensify the effect of the scent. It is possible that this aspect is important, especially for the more night active species such as fallow deer and wild boar, for which sight would potentially be less important. Habitat openness reflects risk and food availability, particularly for forest ungulates. In forest habitat, food availability is generally higher in forest gaps with increased light availability and forest ungulates preferentially feed in these forest gaps (Kuijper et al. 2009). Therefore, spatial distribution of food availability likely interacts with predation risk (Schmidt & Kuijper 2015). However, in our study design food availability was standardized across the habitat openness gradient through the provision of large amounts of feed at each site. Hence, food availability did not confound predation risk in our study.

Multipredator systems often include contrasting risks created by predators with different hunting techniques, suggesting that selection of more open habitat by prey to avoid one predator may actually increase risk created by another predator. For example, Lone et al. (2014) showed that roe deer live in a complex landscape of fear where predation risk by lynx was highest in closed habitats while human hunting created highest risk in open habitats. Importantly, the fact that, in our study, brown bear scent reduced prey species' use of areas with denser vegetation may be maladaptive in areas with high human activity due to the increased risk of human‐caused mortality in more open habitats (Lone et al. 2014). However, the role of human activity is complex, as many studies have shown that human settlements may act as refuge areas where ungulates find protection from large carnivores (the so‐called “human shield” effect, Berger 2007). Future research on the nature of contrasting risk effects between different predatory types (e.g., ambushing lynx vs. chasing wolf) and large carnivores and humans in human‐modified landscapes is imperative, because most wildlife populations reside in, or depend on, regions outside protected areas. Roe deer and wild boar did not show as clear responses to avoid predator scent as fallow deer. Generally, wild boars are relatively unresponsive to predation risk, likely because they are not a primary prey species of European large carnivores (Kuijper et al. 2014; Wikenros et al. 2015). The lack of a strong response by roe deer is more difficult to explain but may be partly due to our relatively small sample size for this species (only 1/3 of the sample size of fallow deer). However, the effect of habitat openness was similar for all species. Therefore, our results indicate that prey naivety is not present in our study area. This reflects other recent studies that showed that prey maintained antipredator responses to their predators that went extinct over a century ago (Li et al. 2011; Chamaillé‐Jammes et al. 2014). The main explanation for prey maintaining these responses is that the innate mechanism of recognition of dangerous predators is an evolutionary response (Chamaillé‐Jammes et al. 2014). Traits with simple reaction norms and direct effects on survival are likely to experience strong, directional selection which may effectively drive the trait to fixation in a population, explaining why it is retained even in the absence of selective pressure. Indeed, Ferrero et al. (2011) recently showed that, contrary with noncarnivores, a large range of carnivores produce large amounts of the exact same chemical in their urine and that this chemical elicits antipredator responses in prey. Predator avoidance by prey species without prior experience to the predator is important, because innate antipredator responses will likely reduce the risk of prey populations suffer from high predation rates due to prey naivety if predators return (Berger et al. 2001).

Innate responses to novel scents, not just carnivore scents, may have been beneficial during the course of evolution (Barks and Godin 2013). To control for the possible effects of novel scent we added reindeer scent as additional “novel scent” control. Without such control scents, prey responses to introduced carnivore scents could simply be due to the novelty and not the actual carnivore cue. However, we did not detect a clear response to reindeer scent in our study area. Nevertheless, we stress the importance of including nonpredator control scents when studying prey response to predator cues to avoid overestimating risk effects on prey, particularly when looking at effects of locally extinct, or recently recolonizing, carnivores.

In conclusion, we have experimentally demonstrated that ungulates reduce their visitation of forest habitats with signs of recent predator presence but that the strength of this response declines with increasing human‐created openness of the forest habitat. Human alterations to forested landscapes allow ungulates to change habitat selection in ways that are not possible in undisturbed forests. Interestingly, however, predators will likely use the altered landscape heterogeneity to their advantage. Thus, predator**–**prey interactions may develop in human‐modified landscapes in novel directions that are yet to be explored, which is highly relevant when multiple large carnivore species are recolonizing former ranges that are now heavily impacted upon by humans (Chapron et al. 2014). In many regions, it is often the carnivore and not the prey that is of conservation concern; however, any negative effect on popular game species can be crucial for the public acceptance of large carnivores (Roskaft et al. 2007; Gangaas et al. 2013). Understanding risk behavior in pristine environments is important to assess what we are potentially losing when natural ecosystems are affected by anthropogenic change. However, future research should focus on human‐modified regions to understand the predator**–**prey interactions actually present in these landscapes. The current wildlife comeback in Europe makes this particularly relevant.

## Data Accessibility

Data will be archived in the public archive Dryad (http://datadryad.org/).

## Conflict of Interest

None declared.
